# PPI versus Histamine H2 Receptor Antagonists for Prevention of Upper Gastrointestinal Injury Associated with Low-Dose Aspirin: Systematic Review and Meta-analysis

**DOI:** 10.1371/journal.pone.0131558

**Published:** 2015-07-06

**Authors:** Chen Mo, Gang Sun, Yan-Zhi Wang, Ming-Liang Lu, Yun-Sheng Yang

**Affiliations:** 1 Institute of Digestive Diseases, Chinese PLA General Hospital, Beijing 100853, China; 2 Cadre Ward No. 2, the General Hospital of Chinese Armed Force Police, Beijing 100039, China; University Hospital Llandough, UNITED KINGDOM

## Abstract

This study compared proton pump inhibitors (PPIs) and histamine H2 receptor antagonists (H_2_RAs) for prevention of low-dose aspirin (LDA)-related gastrointestinal (GI) erosion, ulcer and bleeding. Electronic databases including PubMed, Embase, Cochrane Central Register of Controlled Trials, Chinese National Knowledge Infrastructure, Chinese Biomedical Literature Database, and WanFang Data were searched from the date of their establishment to December 31, 2013. Randomized controlled trials comparing PPIs and H_2_RAs for prevention of GI injury associated with low-dose aspirin (LDA) were collected. Two reviewers independently abstracted studies and patient characteristics and appraised study quality using the Cochrane risk-of-bias tool. Meta-analysis was performed using RevMan 5.1 software. We included nine RCTs involving 1047 patients. The meta-analysis showed that PPIs were superior to H_2_RAs for prevention of LDA-associated GI erosion/ulcer [odds ratio (OR=0.28, 95% confidence interval (CI): 0.16–0.50] and bleeding (OR=0.28, 95% CI: 0.14–0.59). In conclusion, PPIs were superior to H_2_RAs for prevention of LDA-related GI erosion/ulcer and bleeding. Higher quality, large, multicenter RCTs are needed to demonstrate the preventive effect of the two acid-suppressive drugs.

## Introduction

### Rationale

Low-dose aspirin (LDA) is usually defined as 75–325 mg daily. The mechanism of gastrointestinal (GI) injuries associated with LDA can be subdivided into topical and systemic effects. With the widespread use of LDA in primary and secondary prevention of cardiovascular and cerebrovascular diseases, the incidence of LDA-related upper GI injuries, including gastric mucosal erosion, peptic ulcer and bleeding, has increased annually. A retrospective study found that <50% of patients who were long-term LDA users were taking concomitant gastrointestinal protective drugs [1]. Researchers have also found that physicians have poor awareness of LDA-induced GI damage [2], so the prevention of LDA-associated GI injuries has been an important topic for cardiologists and gastroenterologists.

### Objectives

It is well known that proton pump inhibitors (PPIs) reduce the incidence of LDA-associated GI ulcers and bleeding [3–7]. However, concerns about PPI–clopidogrel interaction, overprescribing of PPIs [8] and side effects of PPIs [9–11] have increased in recent years. Histamine H2 receptor antagonists (H_2_RAs) are more cost-effective and safer compared with PPIs. Taha et al. confirmed that standard doses of famotidine decrease LDA-associated GI injuries and suggested that high-dose H_2_RAs are an alternative to PPIs to prevent LDA-associated GI bleeding [12]. Rostom et al. pointed out in their systematic review that PPIs were superior to H_2_RAs for prevention of nonsteroidal anti-inflammatory drug (NSAID)-induced gastroduodenal ulcer [13].

Only a few studies have investigated prevention of LDA-associated GI ulcers and bleeding, and it has not been established whether H_2_RAs are a rational alternative to PPIs. The present meta-analysis compared the effect of PPIs and H_2_RAs for prevention of LDA-related upper GI injuries, and attempted to provide the best evidence for clinical decision making.

## Methods

The reporting format of this systematic review was based on the Preferred Reporting Items for Systematic Reviews and Meta-Analyses (PRISMA) Statement revised in 2009 [14].

### Eligibility criteria

Inclusion criteria. (1) The design of studies was randomized controlled trials. (2) Patients eligible for inclusion were adults (aged ≥18 years) who used LDA for at least two continuous weeks. Studies were included regardless of the patient’s concomitant medication, medical condition and comorbidity. (3) Intervention measures: oral PPIs were used in the experimental group and H_2_RAs were used as the control drugs. (4) Outcomes of studies: the incidence of LDA-related peptic ulcer and upper GI bleeding in the two groups was observed no matter which was primary endpoint or second endpoint. Exclusion criteria: non-randomized clinical trials, cohort studies, case–control studies, pharmacokinetic experiments, and case reports.

### Search

We conducted a comprehensive literature search of PubMed, Embase, Cochrane Central Register of Controlled Trials (CENTRAL), Chinese National Knowledge Infrastructure (CNKI), WanFang Data and Chinese Biomedical Literature Database (CBM) from their inception to December 31, 2013. Only studies published in English and Chinese were included. The search terms included combinations of the following keywords: aspirin, acetylsalicylic, low-dose aspirin, LDA, proton pump inhibitor, PPI, esomeprazole, pantoprazole, omeprazole, rabeprazole, lansoprazole, histamine receptor antagonist, H_2_RA, famotidine, ranitidine, cimetidine, nizatidine, roxatidine, and randomized controlled trial. The search strategy for PubMed as an example is presented below.

#1 aspirin OR acetylsalicylic OR low-dose aspirin OR LDA
#2 proton pump inhibitor OR PPI OR omeprazole OR esomeprazole OR lansoprazole OR pantoprazole OR rabeprazole
#3 histamine receptor antagonist OR H_2_RA OR famotidine OR ranitidine OR cimetidine OR nizatidine OR roxatidine
#4 #1 AND #2 AND #3

### Study selection

Two independent reviewers (C Mo and YZ Wang) used a predefined relevance criteria form to screen the studies. After reading the title and abstract, the documents that did not meet the inclusion criteria and duplicate articles were eliminated. The full text of relevant articles was screened for inclusion. Discrepancies at any stage were resolved by discussion with a third reviewer (G Sun). The level of agreement during screening was evaluated using a κ statistic and we determined *a priori* that an acceptable level of agreement should be at least 0.60.

### Data collection process

The data were extracted after the full text reading. Two independent reviewers (C Mo and YZ Wang) extracted the data. A third independent reviewer (G Sun) reviewed the data abstraction and resolved any discrepancies. When multiple publications reported data from the same population, the trial reporting the primary outcome of interest was considered the major publication. The extracted data included: authors and publication year, medical condition or risk factor, sample size, intervention measures, drug doses, course of treatment, drug co-administration, GI ulcer/erosion or bleeding events, and statistical methods.

### Risk of bias in individual studies

Risk of bias in individual studies was assessed using the Cochrane Risk of Bias tool. This tool assesses the following six domains of bias: sequence generation (decided as low risk, high risk and unclear risk), allocation concealment (decided as low risk, high risk and unclear risk), blinding of outcome assessment (decided as low risk, high risk, and unclear risk), incompleteness outcome data (decided as low risk, high risk and unclear risk), selective outcome reporting (decided as low risk, high risk and unclear risk), and other types of bias (decided as low risk, high risk and unclear risk). The two reviewers (C Mo and YZ Wang) assessed study quality independently and the assessments were verified by the third reviewer (G Sun).

### Statistical analysis

All analyses were conducted using Review Manager version 5.1. For dichotomous data, summary statistics were expressed as odds ratio (OR) with 95% confidence interval (CI) for interpretation. Statistical significance level was considered as α = 0.05. Statistical heterogeneity in the included studies was examined using *I* ^2^ statistics. If the result of the heterogeneity test was *P*≥0.10, a fixed-effect model was used for the meta-analysis; if *P*<0.10, the sources of heterogeneity were investigated. If no obvious clinical heterogeneity and no clear statistical heterogeneity occurred, a random-effect model was used for the meta-analysis. If the clinical heterogeneity was too large, data synthesis should be abandoned and a single research analysis should be used instead. Sensitivity analysis was performed on some of the results of aggregate analysis. Potential publication bias was evaluated by funnel plot analysis.

## Results

### Study selection

**Fig 1:**
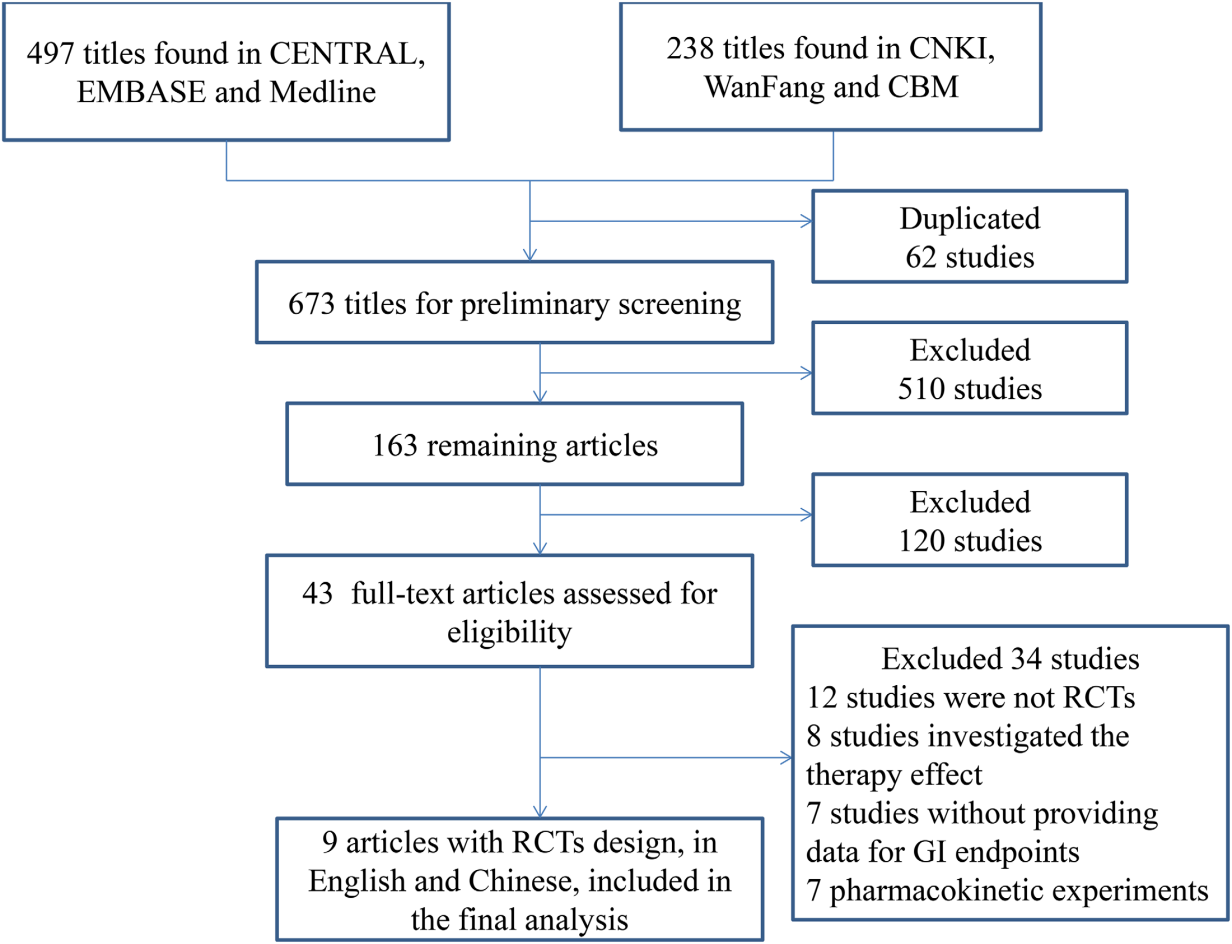
Flow chart of retrieved articles.

The literature search identified 735 articles: 497 published in English and 238 in Chinese. Five hundred and seventy-two articles were excluded because they were duplicate publications or did not meet the inclusion criteria. One hundred and twenty articles were excluded after reading the titles and abstracts. Forty-three full-text articles were retrieved, including 34 articles published in English and nine in Chinese. Twelve articles were excluded because they were not RCTs[15–26]; eight because they compared the therapeutic effects[18,27–33]; seven because they did not investigate upper GI endpoints[34–40]; and seven because they were pharmacokinetic experiments[41–47]. The details of References to Studies Excluded in meta-analysis please see in Supporting Information (S1 File). Nine RCTs fulfilled the inclusion criteria including three in English [48–50] and six in Chinese [51–56]. Fig 1 shows the flow chart of the retrieved articles. The level of agreement between the two reviewers was acceptable (*κ* = 0.67).

### Study characteristics

All the studies included were published in the US or China between 2009 and 2013. Demographic and clinical characteristics of the studies included in this meta-analysis are summarized in Table 1. The number of participants in the experimental group ranged from 42 to 163 and the duration of follow-up from 4 to 52 weeks. The PPIs examined were pantoprazole, rabeprazole, esomeprazole, omeprazole and lansoprazole, at doses ranging from 10 to 40 mg/day. The number of participants in the control group ranged from 22 to 148 and the duration of follow-up from 4 to 52 weeks. The H_2_RAs in the control group included famotidine (20–80 mg/day) and ranitidine (300 mg/day). The risk factors of patients differed among the RCTs. One RCT included patients with peptic ulcer or erosions [48]; one RCT included patients who were negative for *Helicobacter pylori* and without a history of ulcer bleeding or active ulcers [52]; four RCTs included patients with acute coronary syndrome or myocardial infarction [49,50,55,56]; and one RCT included older patients who needed long-term LDA treatment [52]. Three RCTs had endoscopy before and after treatment [48,49,53]. Four RCTs included patients who co-administered clopidogrel and enoxaparin or another anticoagulant [49,50,55,56].

**Table 1:** Characteristics of studies included in the meta-analysis.

				Prevention Group					Control Group				
Studies	Risk Factor	Co-administration	Course	n	Drug	Usage	Ulcer/ Erosion(%)	Bleeding (%)	n	Drug	Usage	Ulcer/ Erosion(%)	Bleeding (%)
Ng 2010[48]	Ulcer or erosions	no	48w	65	pantoprazole	20 mg bid	0 (0)	0 (0)	65	famotidine	40mg bid	8 (12.3)	5 (7.7)
Ng 2012[49]	ACS or MI	Clopidogrel and anticoagulant	4-52w	163	esomeprazole	20 mg qd	1 (0.6)	3 (1.8)	148	famotidine	40 mg qd	6 (4.1)	12 (8.1)
Yano 2012[50]	ACS	Clopidogrel	12m	65	Omeprazole	10 mg qd	-	3(4.6)	65	famotidine	20 mg qd	-	1(1.5)
Guo M 2009[51]	Not clear	no	90d	42	Omeprazole or esomeprazole	20 mg qd	6 (14.3)	-	22	famotidine	20 mg bid	5 (22.7)	-
Sun RR 2012[52]	Elders	no	90d	40	rabeprazole	20 mg qd	3 (7.5)	0 (0)	40	ranitidine	150mg bid	11 (27.5)	1(2.5)
Wang YP 2012[53]	HP-, no ulcer history	no	90d	23	lansoprazole	30 mg qd	2 (8.7)	0 (0)	22	famotidine	20 mg bid	6 (27.3)	1(4.5)
Hu L 2012[54]	Not clear	no	90d	50	rabeprazole	10 mg qd	5(10)	-	48	famotidine	20 mg bid	9 (18.8)	-
Lu BJ 2013[55]	ACS	Clopidogrel	30d	50	omeprazole	40mg qd	-	2(4)	50	ranitidine	150 mg bid	-	9(18)
Wang J 2012[56]	ACS	Clopidogrel	90d	43	esomeprazole	20mg bid	3 (7.0)	-	46	famotidine	20 mg bid	5 (10.9)	-

ACS:acute coronary syndrome; MI:myocardial infarction

### Risk of bias across studies

**Fig 2:**
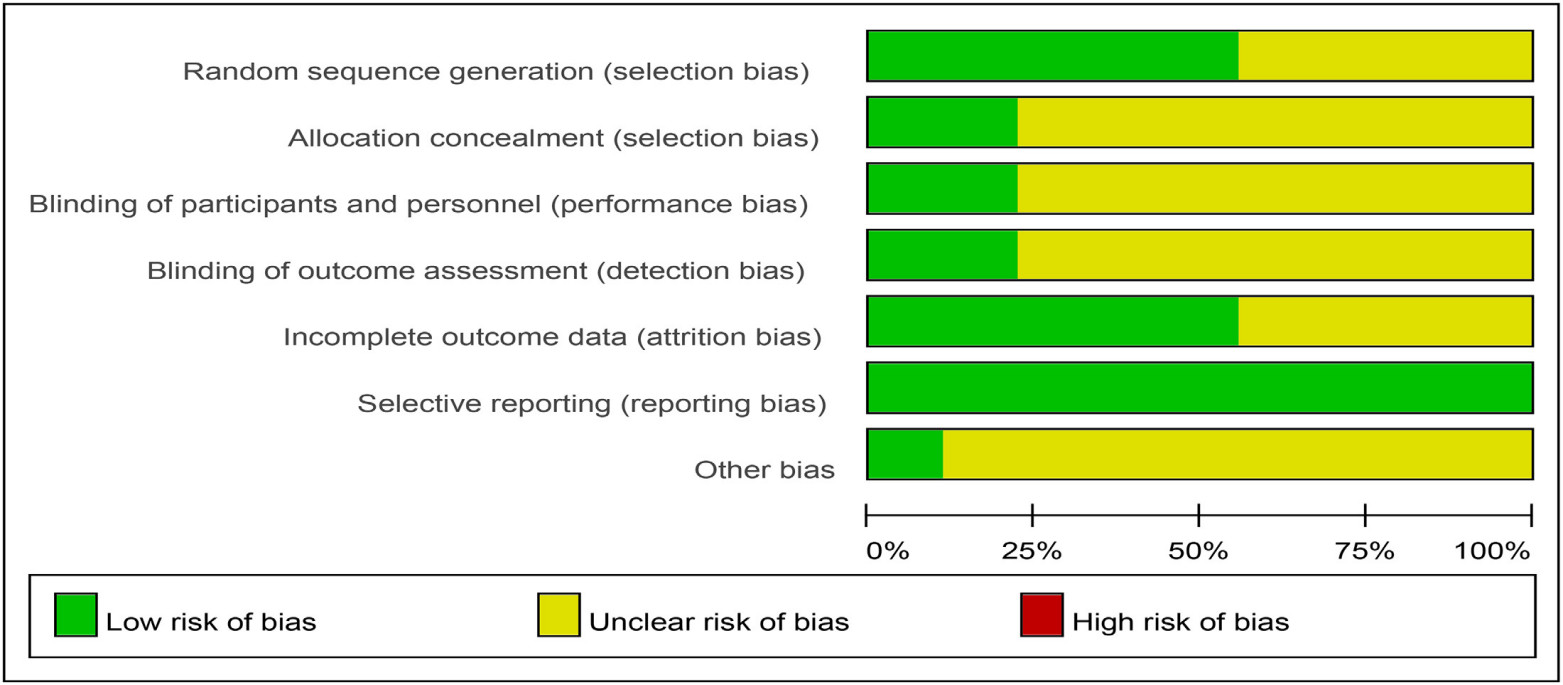
Risk of bias graph.

**Fig 3:**
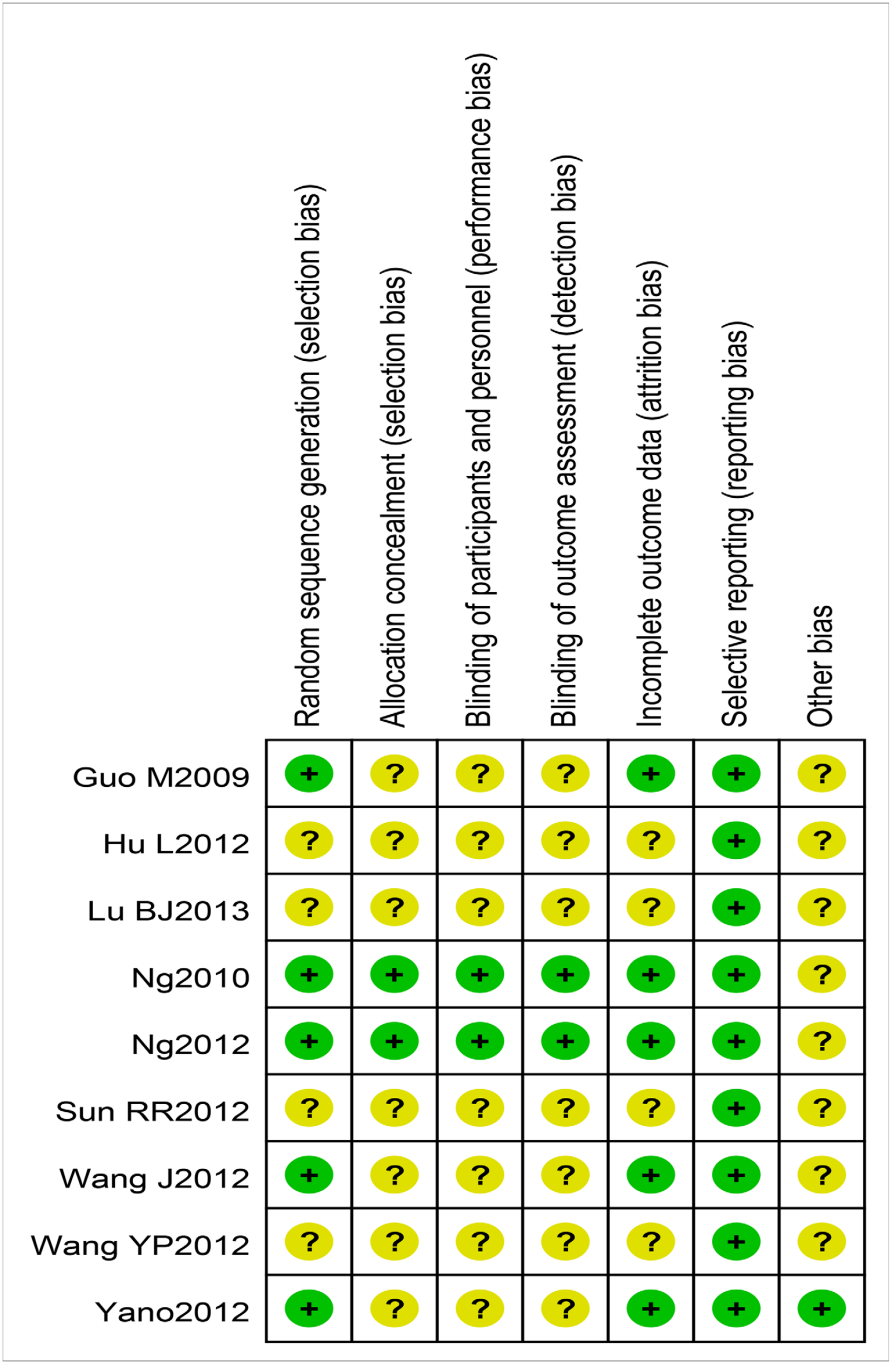
Risk of bias summary.

The risk of bias within the eight studies included in the meta-analysis is summarized in Table 2. Figs 2 and 3 show the risk of bias graph and the risk of bias summary.

**Table 2:** Bias risk evaluation of studies included in the meta-analysis.

Studies	Random sequence generation	Allocation concealment	Blinding	Incomplete outcome data	Selective reporting	Other bias
Guo M 2009 [51]	Low risk	Unclear risk	Unclear risk	Low risk	Low risk	Unclear risk
Ng 2010 [48]	Low risk	Low risk	Low risk	Low risk	Low risk	Unclear risk
Yano 2012[50]	Low risk	Unclear risk	Unclear risk	Low risk	Low risk	Low risk
Wang YP 2012 [53]	Unclear risk	Unclear risk	Unclear risk	Unclear risk	Low risk	Unclear risk
Sun RR 2012 [52]	Unclear risk	Unclear risk	Unclear risk	Unclear risk	Low risk	Unclear risk
Ng 2012 [49]	Low risk	Low risk	Low risk	Low risk	Low risk	Unclear risk
Hu L 2012 [54]	Unclear risk	Unclear risk	Unclear risk	Unclear risk	Low risk	Unclear risk
Wang J 2012 [56]	Low risk	Unclear risk	Unclear risk	Low risk	Low risk	Unclear risk
Lu BJ 2013 [55]	Unclear risk	Unclear risk	Unclear risk	Unclear risk	Low risk	Unclear risk

#### Comparison of incidence of LDA-associated GI ulcers/erosions

**Fig 4:**
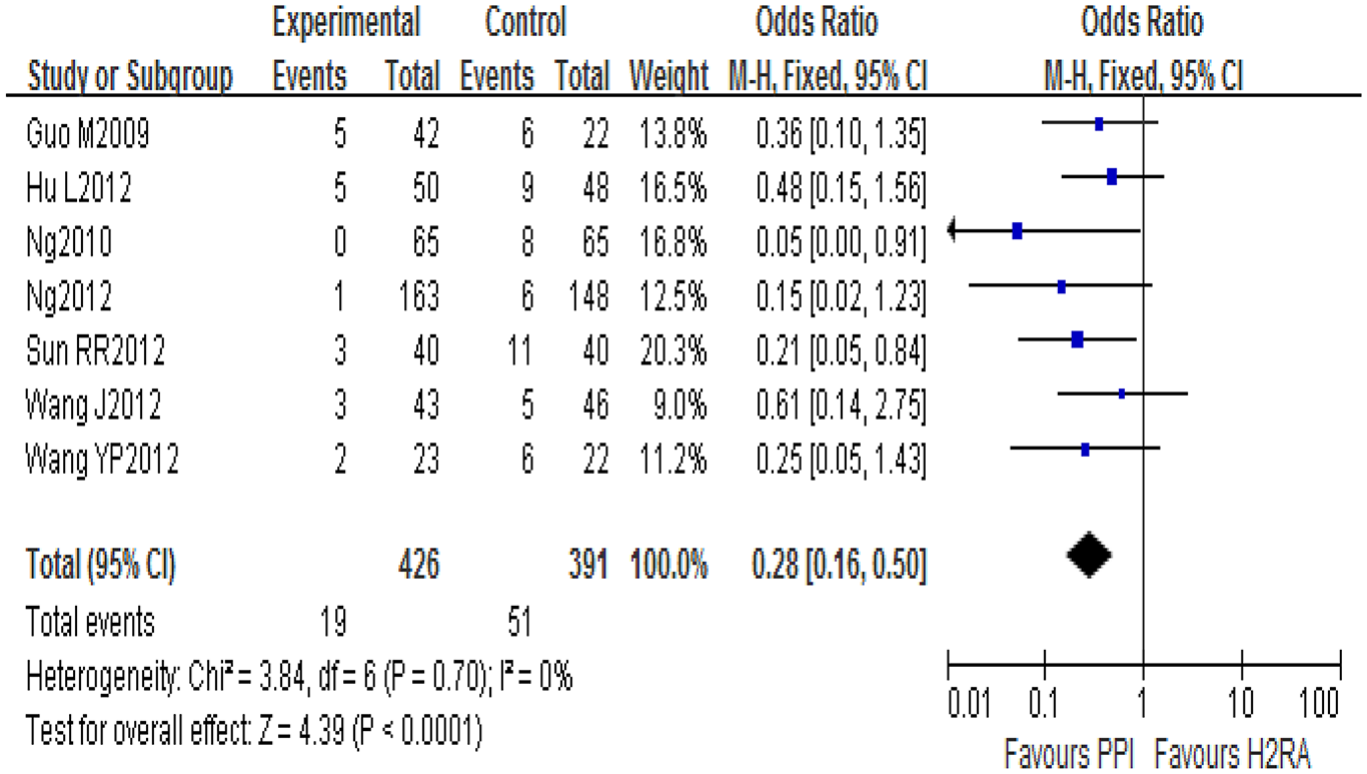
H_2_RAs and PPIs for prevention of LDA-associated GI ulcers/erosions.

Seven of the eight included studies reported the incidence of LDA-associated GI ulcer/erosions in the PPI and H_2_RA groups. There was no statistical heterogeneity among the results (*I* ^2^ = 0, *P* = 0.70), so a fixed-effect model was used for meta-analysis. The result showed that PPIs were superior to H_2_RAs (OR = 0.28, 95% CI: 0.16–0.50) for prevention of LDA-associated GI ulcers or erosions (Fig 4).

#### Comparison of incidence of LDA-associated GI bleeding

**Fig 5:**
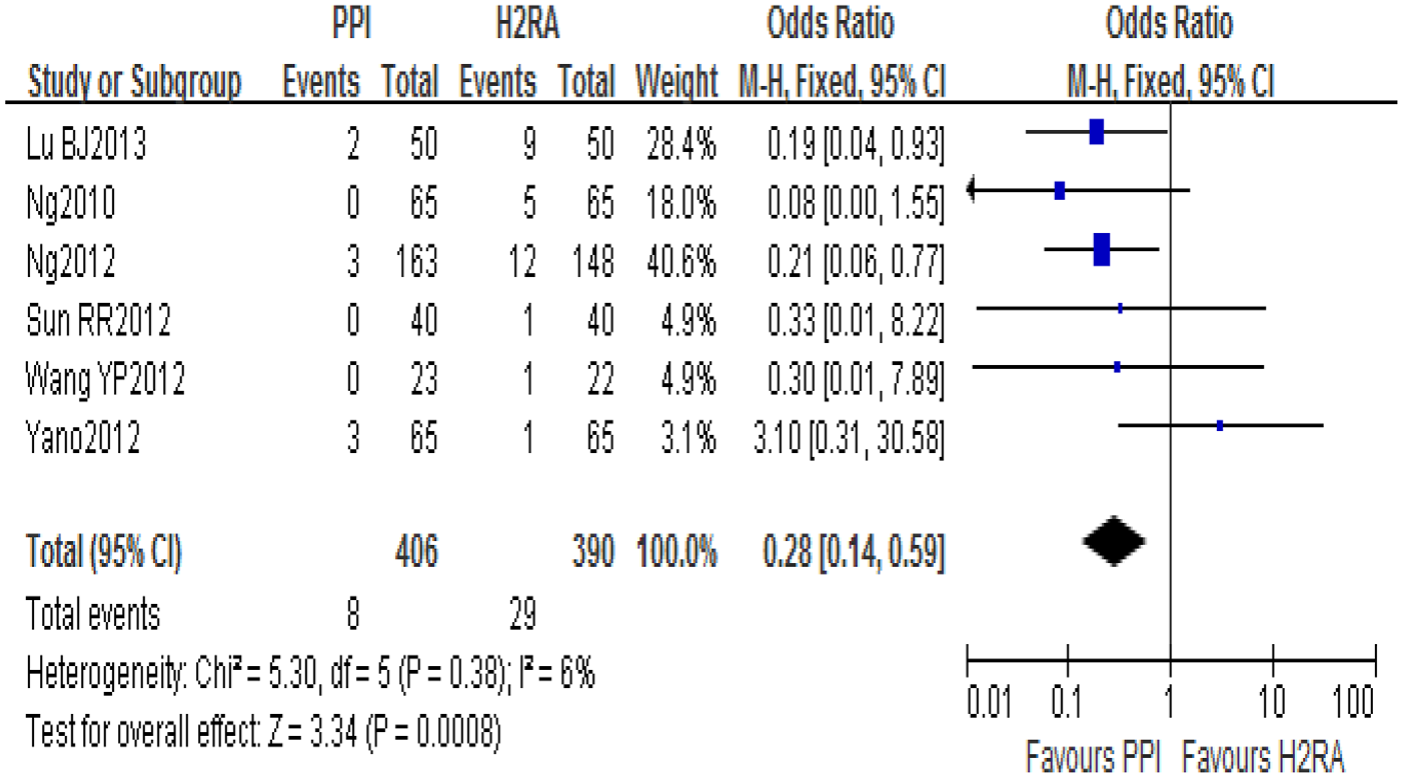
PPIs and H_2_RAs for prevention of LDA-associated GI bleeding.

Six of the eight included studies reported the incidence of LDA-associated GI bleeding in the PPI and H_2_RA groups. There was no statistical heterogeneity among the results (*I* ^2^ = 6%, *P* = 0.38) and a fixed-effect model was used for meta-analysis. The result showed that PPIs were superior to H_2_RAs (OR = 0.28 95%CI: 0.14–0.59) for prevention of LDA-associated GI bleeding (Fig 5).

### Publication bias

**Fig 6:**
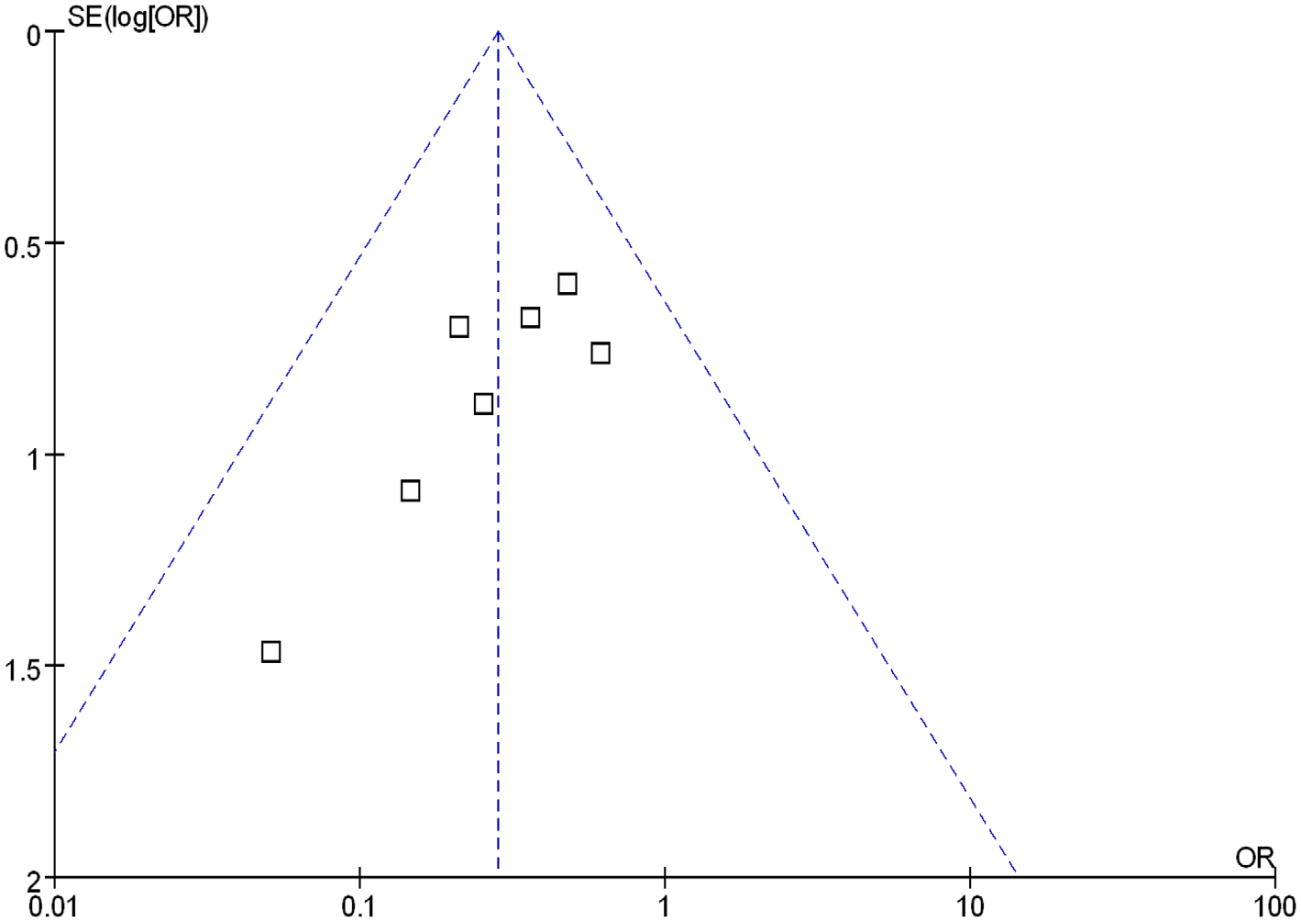
Funnel plot analysis of the trials of H_2_RAs and PPIs for prevention of LDA- associated ulcers/erosions.

Funnel plot analysis of the seven RCTs of PPIs and H_2_RAs for prevention of LDA-associated GI ulcers/erosions indicated an asymmetrical distribution that indicated the presence of publication bias (Fig 6).

## Discussion

### Summary of evidence

It is well known that long-term use of LDA increases the risk of upper GI injuries and bleeding [57]. The pathogenetic mechanism involves topical effects of acid reverse diffusion and systemic effects of inhibiting prostaglandin synthesis through the cyclo-oxygenase-1 pathway. Anti-secretory drugs are effective in reducing upper GI mucosal injuries and bleeding complications by inhibiting gastric acid secretion and lowering gastric pH. Studies of prophylaxis for LDA-associated GI injuries have focused on PPIs, but PPIs are expensive and their long-term use has side effects such as *Clostridium-difficile*-associated diarrhea, community-acquired pneumonia, osteoporosis and fractures [9–11]. LDA is frequently prescribed concurrently with clopidogrel in dual antiplatelet therapy. However, PPI–clopidogrel interaction increases cardiovascular risks so the use of PPI with LDA is currently restricted. As traditional anti-secretory drugs, H_2_RAs are cost-effective. The American College of Cardiology Foundation (ACCF), American College of Gastroenterology (ACG) and American Heart Association (AHA) revised the expert consensus document of 2010 on reducing the GI risks of antiplatelet therapy and NSAID use, which states that H_2_RAs are a reasonable alternative to PPIs for the prophylaxis and treatment of LDA-associated GI injury [58]. However, the preventive effect of H_2_RAs is still controversial. The OITA-GF2 study indicated that lansoprazole might be more effective than famotidine in preventing the development of LDA-related gastroduodenal injuries [59]. Ng et al. concluded that H_2_RAs were inferior to PPIs for preventing LDA-related upper GI injuries in a cohort study and RCTs [48,49,60]. The present meta-analysis compared the preventive effect of PPIs and H_2_RAs in LDA-associated GI injuries, and explored the advantages of H_2_RAs, so as to provide more reasonable and cost-effective drugs for clinical practice.

There were nine RCTs included in our meta-analysis. We found that PPIs were superior to H_2_RAs for prevention of LDA-associated upper GI ulcers/erosions and bleeding. However, among the nine RCTs included, six studies were from mainland China and had small samples and were poorly reported. The bias of random sequence generation, allocation concealment, blinding method, incomplete outcome data, and other bias in most of the studies were not clear, which means that the results of our meta-analysis should be interpreted with caution.

Lanas et al. discovered in a case–control study that *H*. *pylori* infection increased the risk of GI bleeding (OR, = 4.7; 95% CI: 2.0–10.9) and indicated that *H*. *pylori* infection was an independent risk factor for LDA-associated GI bleeding [61]. Only one RCT in our analysis included patients who were negative for *H*. *pylori*, and one RCT showed *H*. *pylori* eradication. Most of our studies did not determine *H*. *pylori* infection status, thus, it is not clear whether infection interacted with LDA to increase mucosal injuries. So, selection bias may have been present in our meta-analysis.

### Limitations

Because meta-analyses are secondary research, their conclusions are influenced by the quality of the included studies. There were some limitations in our meta-analysis that should be mentioned. First, the quality of some studies was low and we need more high-quality, multicenter, high-standard RCTs in the future. Second, we searched the unpublished articles in English and Chinese, but were unable to identify all relevant unpublished data to include. Funnel plot analysis showed that publication bias was also present. Third, we searched articles written in English and Chinese, but only three RCTs in English were included, and they may have had reporting bias. Last, All of the nine trials included Asian patients, one from Japan, two from Hong Kong and the others from mainland China. So the representativeness of patients are not well enough.

## Conclusions

In conclusion, PPIs were superior to H_2_RAs in preventing LDA-associated GI ulcers/erosions and bleeding. Some of the RCTs included in our meta-analysis were poorly reported and of low quality, therefore, our meta-analysis should be interpreted with caution. More multicenter, high-quality RCTs are needed to compare two anti-secretory drugs for prevention of LDA-associated GI injuries.

## Supporting Information

S1 File References to Studies Excluded in Meta-analysis. (PDF)

S2 File PRISMA Statement. (PDF)

S1 PRISMA Checklist PRISMA 2009 Checklist. (DOC)

## References

[1] Luk HH. Use of gastroprotective drugs in patients receiving low-dose aspirin. J Chin Med Assoc.2009; 72: 356–361. 10.1016/S1726-4901(09)70387-9 19581141

[2] Zhu LL, Xu LC, Chen Y, Zhou Q, Zeng S. Poor awareness of preventing aspirin-induced gastrointestinal injury with combined protective medications. World J Gastroenterol.2012; 18: 3167–3172. 10.3748/wjg.v18.i24.3167 22791953 PMC3386331

[3] Lai KC, Lam SK, Chu KM, Wong BC, Hui WM, Hu WH, et al Lansoprazole for the prevention of recurrences of ulcer complications from long-term low-dose aspirin use. N Engl J Med.2002; 346: 2033–2038. 12087138 10.1056/NEJMoa012877

[4] Yeomans N, Lanas A, Labenz J, van Zanten SV, van Rensburg C, Racz I, et al Efficacy of esomeprazole (20 mg once daily) for reducing the risk of gastroduodenal ulcers associated with continuous use of low-dose aspirin. Am J Gastroenterol.2008; 103: 2465–2473. 10.1111/j.1572-0241.2008.01995.x 18637091

[5] Sugano K, Matsumoto Y, Itabashi T, Abe S, Sakaki N, Ashida K, et al Lansoprazole for secondary prevention of gastric or duodenal ulcers associated with long-term low-dose aspirin therapy: results of a prospective, multicenter, double-blind, randomized, double-dummy, active-controlled trial. J Gastroenterol.2011; 46: 724–735. 10.1007/s00535-011-0397-7 21499703 PMC3117278

[6] Scheiman JM, Devereaux PJ, Herlitz J, Katelaris PH, Lanas A, Veldhuyzen van Zanten S, et al Prevention of peptic ulcers with esomeprazole in patients at risk of ulcer development treated with low-dose acetylsalicylic acid: a randomised, controlled trial (OBERON). Heart.2011; 97: 797–802. 10.1136/hrt.2010.217547 21415072 PMC3088470

[7] Sanuki T, Fujita T, Kutsumi H, Hayakumo T, Yoshida S, Inokuchi H, et al Rabeprazole reduces the recurrence risk of peptic ulcers associated with low-dose aspirin in patients with cardiovascular or cerebrovascular disease: a prospective randomized active-controlled trial. J Gastroenterol.2012; 47: 1186–1197. 10.1007/s00535-012-0588-x 22526273

[8] Forgacs I, Loganayagam A. Overprescribing proton pump inhibitors. BMJ.2008; 7634:2–3. 10.1136/bmj.39406.449456.BE PMC2174763 18174564

[9] Garcia Rodriguez LA, Ruigomez A, Panes J. Use of acid-suppressing drugs and the risk of bacterial gastroenteritis. Clin Gastroenterol Hepatol. 2007; 5: 1418–1423. 18054750 10.1016/j.cgh.2007.09.010

[10] Ramsay EN, Pratt NL, Ryan P, Roughead EE. Proton pump inhibitors and the risk of pneumonia: a comparison of cohort and self-controlled case series designs. BMC Med Res Methodol.2013; 13: 82 10.1186/1471-2288-13-82 23800078 PMC3699413

[11] Fraser LA, Leslie WD, Targownik LE, Papaioannou A, Adachi JD; CaMos Reserch Group. The effect of proton pump inhibitors on fracture risk: report from the Canadian Multicenter Osteoporosis Study. Osteoporos Int. 2013; 24: 1161–1168. 10.1007/s00198-012-2112-9 22890365 PMC5096922

[12] Taha AS, McCloskey C, Prasad R, Bezlyak V. Famotidine for the prevention of peptic ulcers and oesophagitis in patients taking low-dose aspirin (FAMOUS): a phase III, randomised, double-blind, placebo-controlled trial. Lancet. 2009; 374: 119–125. 10.1016/S0140-6736(09)61246-0 19577798

[13] Rostom A, Dube C, Wells G, Tugwell P, Welch V, Jolicoeur E, et al Prevention of NSAID-induced gastroduodenal ulcers. Cochrane Database Syst Rev. 2002;4: CD002296 12519573 10.1002/14651858.CD002296

[14] Moher D, Liberati A, Tetzlaff J, Altman DG; PRISMA Group. Preferred reporting items for systematic reviews and meta-analyses: the PRISMA statement. BMJ. 2009; 21: b2700. PMC3090117 21603045

[15] Lin KJ, Hernandez-Diaz S, Garcia Rodriguez LA. Acid suppressants reduce risk of gastrointestinal bleeding in patients on antithrombotic or anti-inflammatory therapy. Gastroenterology. 2011; 141: 71–79. 10.1053/j.gastro.2011.03.049 21458456

[16] Yasuda H, Yamada M, Sawada S, Endo Y, Inoue K, Asano F et al Upper gastrointestinal bleeding in patients receiving dual antiplatelet therapy after coronary stenting. Intern Med. 2009; 48: 1725–1730. 19797827 10.2169/internalmedicine.48.2031

[17] Tamura A, Murakami K, Kadota J. Prevalence of gastroduodenal ulcers/erosions in patients taking low-dose aspirin with either 15 mg/day of lansoprazole or 40 mg/day of famotidine: the OITA-GF study 2. BMC Res Notes. 2013; 6: 1756–0500. 10.1186/1756-0500-6-116 PMC3626555 23531145

[18] Nakashima S, Ota S, Arai S, Yoshino K, Inao M, Ishikawa K et al Usefulness of anti-ulcer drugs for the prevention and treatment of peptic ulcers induced by low doses of aspirin. World J Gastroenterol. 2009; 15: 727–731. 19222098 10.3748/wjg.15.727 PMC2653442

[19] Wu CY, Chan FK, Wu MS, Kuo KN, Wang CB, Tsao CR et al Histamine2-receptor antagonists are an alternative to proton pump inhibitor in patients receiving clopidogrel. Gastroenterology. 2010; 139: 1165–1171. 10.1053/j.gastro.2010.06.067 20600012

[20] Lanas A, Garcia-Rodriguez LA, Arroyo MT, Bujanda L, Gomollon F, Forne M et al Effect of antisecretory drugs and nitrates on the risk of ulcer bleeding associated with nonsteroidal anti-inflammatory drugs, antiplatelet agents, and anticoagulants. Am J Gastroenterol. 2007; 102: 507–515. 17338735 10.1111/j.1572-0241.2006.01062.x

[21] Wang Z, Yang XC, Cai J, Chen ML, Wan XH. Proton pump inhibitors on the antiplatelet function of clopidogrel after coronary artery intervention treatment of acute myocardial infarction patients. Chinese Journal of Medicine. 2013; 48: 20–22.

[22] Tian XX, Du H, Zheng YF, Zhou Q, Zhang Y, Bai YL. Esomeprazole Combined with Rebamipide in Preventing Gastric Mucosal Lesions Induced by Non-steroidal Anti-inflammatory Drugs. Chin Gen Pract. 2013; 16: 2407–2409.

[23] Du Y, Wang M, Pan JS. Analysis of acid-suppressive drug choice after PCI in patients with coronary heart diseases. Chin New Drug J. 2013; 22: 975–982.

[24] Ji CY, Wang Y, Tan SY, Liu H. Efficacy of esomeprazole on gastrointestinal bleeding caused by NSAIDs in the eldery. Pract Geriatr. 2011; 25: 128–130.

[25] Wang Y. Effect of different proton pump inhibitors or H2 receptor antagonists on the anti-platelet functions of clopidogrel in patients after PCI. Thesis, Fourth Military Medical University. 2012.

[26] Wu G. Effect of prognosis of patients with proton pump inhibitors after percutaneous coronary intervention treatment: A retrospective analysis. Thesis, Guang Xi Medical University. 2012.

[27] Nema H, Kato M. Comparative study of therapeutic effects of PPI and H2RA on ulcers during continuous aspirin therapy. World J Gastroenterol. 2010; 16: 5342–5346. 21072898 10.3748/wjg.v16.i42.5342 PMC2980684

[28] Sakurada T, Kawashima J, Ariyama S, Kani K, Takabayashi H, Ohno S et al Comparison of adjuvant therapies by an H2-receptor antagonist and a proton pump inhibitor after endoscopic treatment in hemostatic management of bleeding gastroduodenal ulcers. Dig Endosc. 2012; 24: 93–99. 10.1111/j.1443-1661.2011.01176.x 22348833

[29] Sakurai K, Nagahara A, Inoue K, Akiyama J, Mabe K, Suzuki J et al Efficacy of omeprazole, famotidine, mosapride and teprenone in patients with upper gastrointestinal symptoms: an omeprazole-controlled randomized study (J-FOCUS). BMC Gastroenterol. 2012; 12: 12–42. 22548767 10.1186/1471-230X-12-42 PMC3419613

[30] Tanaka S, Nishigaki K, Ojio S, Okubo M, Yasuda S, Ishihara Y et al Can negative cardiac effect of proton pump inhibitor and high-dose H2-blocker have clinical influence on patients with stable angina? J Cardiol. 2008; 52: 39–48. 10.1016/j.jjcc.2008.05.004 18639776

[31] Sener-Muratoglu G, Paskaloglu K, Arbak S, Hurdag C, Ayanoglu-Dulger G. Protective effect of famotidine, omeprazole, and melatonin against acetylsalicylic acid-induced gastric damage in rats. Dig Dis Sci. 2001; 46: 318–330. 11281181 10.1023/a:1005652815921

[32] Cao YZ, Liu L, Wang BL, Lu J, He BJ, Zhong YX, et al PPI vs H2A-Which is stronger inhibitor on acute peptic ulcer bleeding. J Fourth Mil Med Univ. 2001; 22: 790–792.

[33] Li M. High dose proton pump inhibitor and H2 receptor antagonist in the therapy of 86 upper gastrointestinal bleeding patients. Cli Med. 2011; 31:56–57.

[34] Brophy GM, Brackbill ML, Bidwell KL, Brophy DF. Prospective, randomized comparison of lansoprazole suspension, and intermittent intravenous famotidine on gastric pH and acid production in critically ill neurosurgical patients. Neurocrit Care. 2010; 13: 176–181. 10.1007/s12028-010-9397-3 20596795

[35] Kim YJ, Cheon JH, Lee SK, Kim JH, Lee YC. Rebamipide may be comparable to H2 receptor antagonist in healing iatrogenic gastric ulcers created by endoscopic mucosal resection: a prospective randomized pilot study. J Korean Med Sci. 2010; 25: 583–588. 10.3346/jkms.2010.25.4.583 20358002 PMC2844599

[36] Furuta T, Shirai N, Sugimoto M, Nakamura A, Okudaira K, Kajimura M et al Effect of concomitant dosing of famotidine with lansoprazole on gastric acid secretion in relation to CYP2C19 genotype status. Aliment Pharmacol Ther. 2005; 22: 67–74. 15963082 10.1111/j.1365-2036.2005.02523.x

[37] Arbel Y, Birati EY, Finkelstein A, Halkin A, Kletzel H, Abramowitz Y et al Platelet inhibitory effect of clopidogrel in patients treated with omeprazole, pantoprazole, and famotidine: a prospective, randomized, crossover study. Clin Cardiol. 2013; 36: 342–346. 10.1002/clc.22117 23630016 PMC6649548

[38] Nagata Y, Inomata J, Kinoshita M, Kurokawa K, Aburadani I, Maruyama M et al Impact of proton pump inhibitors or famotidine on the antiplatelet actions during dual-antiplatelet therapy in Japanese patients. Cardiovasc Interv Ther. 2013; 28: 22–29. 10.1007/s12928-012-0123-2 22886368

[39] Tunggal P, Ng FH, Lam KF, Chan FK, Lau YK. Effect of esomeprazole versus famotidine on platelet inhibition by clopidogrel: a double-blind, randomized trial. Am Heart J. 2011; 162: 870–874. 10.1016/j.ahj.2011.08.007 22093203

[40] Li Y, Wang ZR. Comparison of famotidine and rabeprazole on anti-platelet function of clopidogrel in patients with coronary stenting. Shaanxi Med J. 2013; 42: 1186–1188.

[41] Nishino M, Sugimoto M, Kodaira C, Yamade M, Uotani T, Shirai N et al Preventive effects of lansoprazole and famotidine on gastric mucosal injury induced by low-dose aspirin in Helicobacter pylori-negative healthy volunteers. J Clin Pharmacol. 2011; 51: 1079–1086. 10.1177/0091270010376194 20663999

[42] Tolbert K, Bissett S, King A, Davidson G, Papich M, Peters E et al Efficacy of oral famotidine and 2 omeprazole formulations for the control of intragastric pH in dogs. J Vet Intern Med. 2011; 25: 47–54. 10.1111/j.1939-1676.2010.0651.x 21143305

[43] Takahashi Y, Amano Y, Yuki T, Ose T, Miyake T, Kushiyama Y et al Influence of acid suppressants on gastric emptying: cross-over analysis in healthy volunteers. J Gastroenterol Hepatol. 2006; 21: 1664–1668. 16984586 10.1111/j.1440-1746.2006.04270.x

[44] Bersenas AM, Mathews KA, Allen DG, Conlon PD. Effects of ranitidine, famotidine, pantoprazole, and omeprazole on intragastric pH in dogs. Am J Vet Res. 2005; 66: 425–431. 15822586 10.2460/ajvr.2005.66.425

[45] Parkman HP, Urbain JL, Knight LC, Brown KL, Trate DM, Miller MA et al Effect of gastric acid suppressants on human gastric motility. Gut. 1998; 42: 243–250. 9536950 10.1136/gut.42.2.243 PMC1726985

[46] Iida H, Inamori M, Akimoto K, Mawatari H, Endo H, Nozaki Y et al Early effects of intravenous administrations of lansoprazole and famotidine on intragastric pH. Hepatogastroenterology. 2009; 56: 551–554. 19579641

[47] Sakaguchi M, Ashida K, Umegaki E, Miyoshi H, Katsu K. Suppressive action of lansoprazole on gastric acidity and its clinical effect in patients with gastric ulcers: comparison with famotidine. J Clin Gastroenterol. 1995; 20: S27–31. 7594334 10.1097/00004836-199506002-00008

[48] Ng FH, Wong SY, Lam KF, Chu WM, Chan P, Ling YH, et al Famotidine Is Inferior to Pantoprazole in Preventing Recurrence of Aspirin-Related Peptic Ulcers or Erosions. Gastroenterology.2010; 138: 82–88. 10.1053/j.gastro.2009.09.063 19837071

[49] Ng FH, Tunggal P, Chu WM, Lam KF, Li A, Chan K, et al Esomeprazole compared with famotidine in the prevention of upper gastrointestinal bleeding in patients with acute coronary syndrome or myocardial infarction. Am J Gastroenterol.2012; 107: 389–396. 10.1038/ajg.2011.385 22108447

[50] Yano H, Tsukahara K, Morita S, Endo T, Sugano T, Hibi K, et al Influence of omeprazole and famotidine on the antiplatelet effects of clopidogrel in addition to aspirin in patients with acute coronary syndromes: a prospective, randomized, multicenter study. Circ J.2012; 76: 2673–2680. 22864179 10.1253/circj.cj-12-0511

[51] Guo M, Wang J, Zou YC, Weng Y. The clinical effect of esomeprazole in prevention of low dose aspirin induced gastric mucosal injury. Chin J Dig.2009; 29: 481–482.

[52] Sun RR, He YQ, Li R. The effect of rabeprazole in prevention of low-dose aspirin induced gastric mucosal injury in 40 elder patients. Chin J Mod Drug. 2012; Appl 06: 79–80.

[53] Wang YP, Wang RJ. The clinical effect of lansoprazole in prevention of low dose aspirin induced gastric mucosal injury. Strait Pharm J 24:114–115.

[54] Hu L, Zhou T, Xia YH. (2012) The effect of rabeprazole in prevention of low dose aspirin induced gastric mucosal injury. MMJC. 2012; 14:100–101.

[55] Lu BJ, Qiao YJ, Yin YD. Omeprazole affects antiplatelet efficacy of clopidogrel and aspirin after intervention therapy. Chin J Prac Med. 2013; 40: 87–88.

[56] Wang J, Zhou GK, Guo M, Zou YC, Wang MX. The efficacy in prevention of upper gastrointestinal injury in patients of acute coronary syndrome with esomeprazole. Chin J Card Res. 2012; 10: 191–195.

[57] Lanas A, Wu P, Medin J, Mills EJ. Low doses of acetylsalicylic acid increase risk of gastrointestinal bleeding in a meta-analysis. Clin Gastroenterol Hepatol.2011; 9: 762–768. 10.1016/j.cgh.2011.05.020 21699808

[58] Abraham NS, Hlatky MA, Antman EM, Bhatt DL, Bjorkman DJ, Clark CB, et al ACCF/ACG/AHA 2010 Expert Consensus Document on the concomitant use of proton pump inhibitors and thienopyridines: a focused update of the ACCF/ACG/AHA 2008 expert consensus document on reducing the gastrointestinal risks of antiplatelet therapy and NSAID use: a report of the American College of Cardiology Foundation Task Force on Expert Consensus Documents. Circulation. 2010; 122: 2619–2633. 10.1161/CIR.0b013e318202f701 21060077

[59] Tamura A, Murakami K, Kadota J. Prevalence of gastroduodenal ulcers/erosions in patients taking low-dose aspirin with either 15 mg/day of lansoprazole or 40 mg/day of famotidine: the OITA-GF study 2. BMC Res Notes. 2013; 6: 1756–0500. 10.1186/1756-0500-6-116 PMC3626555 23531145

[60] Ng FH, Lam KF, Wong SY, Chang CM, Lau YK, Yuen WC, et al Upper gastrointestinal bleeding in patients with aspirin and clopidogrel co-therapy. Digestion. 2008; 77: 173–177. 10.1159/000141264 18577887

[61] Lanas A, Fuentes J, Benito R, Serrano P, Bajador E, Sainz R. Helicobacter pylori increases the risk of upper gastrointestinal bleeding in patients taking low-dose aspirin. Aliment Pharmacol Ther.2002; 16: 779–786. 11929396 10.1046/j.1365-2036.2002.01230.x

