# Supplementary material for: PPI versus Histamine H2 Receptor Antagonists for Prevention of Upper Gastrointestinal Injury Associated with Low-Dose Aspirin: Systematic Review and Meta-analysis
Source: PLoS One. 2015 Jul 6;10(7):e0131558. doi: 10.1371/journal.pone.0131558 (PMC4493004; doi:10.1371/journal.pone.0131558)
Supplement: S1 File — (PDF) [file pone.0131558.s001.pdf]

References to Studies Excluded in Meta-analysis

12 for non-RCT design:

[15] Lin KJ et al 2011.

[16] Yasuda H et al 2009.

[17] Tamura A et al 2013.

[18] Nakashima S et al 2009.

[19] Wu CY et al 2010.

[20] Lanas A et al 2007.

[21] Wang Z et al 2013.

[22] Tian XX et al 2013.

[23] Du Y et al 2013.

[24] Ji CY et al 2011.

[25] Wang Y 2012.

[26] Wu G 2012.

8 for compared the therapeutic effects but not prevention effects:

[18] Nakashima S et al 2009.

[27] Nema H et al 2010.

[28] Sakurada T et al 2012.

[29] Sakurai K et al 2012.

[30] Tanaka S et al 2008.

[31] Sener-Muratoglu G et al 2001.

[32] Cao YZ et al 2001.

[33] Li M 2011.

7 for not investigate upper GI endpoints:

[34] Brophy GM et al 2010.

[35] Kim YJ et al 2010.

[36] Furuta T et al 2005.

[37] Arbel Y et al 2013.

[38] Nagata Y et al 2013.

[39] Tunggal P et al 2011.

[40] Li Y et al 2013.

7 for pharmacokinetic experiments:

[41] Nishino M et al 2011.

[42] Tolbert K et al 2011.

[43] Takahashi Y et al 2006.

[44] Bersenas AM et al 2005.

[45] Parkman HP et al 1998.

[46] Iida H et al 2009.

[47] Sakaguchi M et al 1995.

15. Lin KJ, Hernandez-Diaz S, Garcia Rodriguez LA. Acid suppressants reduce risk of gastrointestinal bleeding in patients on antithrombotic or anti-inflammatory therapy. Gastroenterology 2011; 141: 71-79.

16. Yasuda H, Yamada M, Sawada S et al. Upper gastrointestinal bleeding in patients receiving dual antiplatelet therapy after coronary stenting. Intern Med 2009; 48: 1725-1730.

17. Tamura A, Murakami K, Kadota J. Prevalence of gastroduodenal ulcers/erosions in patients taking low-dose aspirin with either 15 mg/day of lansoprazole or 40 mg/day of famotidine: the OITA-GF study 2. BMC Res Notes 2013; 6: 1756-0500.

18. Nakashima S, Ota S, Arai S et al. Usefulness of anti-ulcer drugs for the prevention and treatment of peptic ulcers induced by low doses of aspirin. World J Gastroenterol 2009; 15: 727-731.

19. Wu CY, Chan FK, Wu MS et al. Histamine2-receptor antagonists are an alternative to proton pump inhibitor in patients receiving clopidogrel. Gastroenterology 2010; 139: 1165-1171.

20. Lanas A, Garcia-Rodriguez LA, Arroyo MT et al. Effect of antisecretory drugs and nitrates on the risk of ulcer bleeding associated with nonsteroidal anti-inflammatory drugs, antiplatelet agents, and anticoagulants. Am J Gastroenterol 2007; 102: 507-515.

21. 王喆, 杨新春, 蔡军 et al. 不同质子泵抑制剂对急性心肌梗死冠状动脉介入治疗术后氯吡格雷抗血小板功能的影响. 中国医刊 2013; 20-22.

22. 田笑笑, 杜浩, 郑玉峰 et al. 埃索美拉唑与瑞巴派特联合使用在预防非甾体

抗炎药相关性胃黏膜损伤中的疗效研究. 中国全科医学 2013; 2407-2409.

23. 杜瑜, 王淼, 潘金水. 冠心病PCI手术患者抑酸药物的选用分析. 中国新药杂志 2013; 975-978+982.

24. 计春燕, 汪毅, 谭诗云, 刘浩. 埃索美拉唑防治非甾体类抗炎药所致老年人上消化道出血的疗效观察. 实用老年医学 2011; 128-130.

25. 王艳. 不同质子泵抑制剂或 H2 受体拮抗剂对冠脉支架术后氯吡格雷抗血小板药效的影响. In. 第四军医大学 2012.

26. 吴刚. 回顾性分析：使用质子泵抑制剂对经皮冠状动脉介入治疗术后患者预后的影响. In. 广西医科大学 2012.

27. Nema H, Kato M. Comparative study of therapeutic effects of PPI and H2RA on ulcers during continuous aspirin therapy. World J Gastroenterol 2010; 16: 5342-5346.

28. Sakurada T, Kawashima J, Ariyama S et al. Comparison of adjuvant therapies by an H2-receptor antagonist and a proton pump inhibitor after endoscopic treatment in hemostatic management of bleeding gastroduodenal ulcers. Dig Endosc 2012; 24: 93-99.

29. Sakurai K, Nagahara A, Inoue K et al. Efficacy of omeprazole, famotidine, mosapride and teprenone in patients with upper gastrointestinal symptoms: an omeprazole-controlled randomized study (J-FOCUS). BMC Gastroenterol 2012; 12: 12-42.

30. Tanaka S, Nishigaki K, Ojio S et al. Can negative cardiac effect of proton pump inhibitor and high-dose H2-blocker have clinical influence on patients with stable angina? J Cardiol 2008; 52: 39-48.

31. Sener-Muratoglu G, Paskaloglu K, Arbak S et al. Protective effect of famotidine, omeprazole, and melatonin against acetylsalicylic acid-induced gastric damage in rats. Dig Dis Sci 2001; 46: 318-330.

32. 曹义战, 刘莉, 王伯良 et al. 两类胃酸分泌抑制剂治疗急性消化性溃疡出血的比较研究. 第四军医大学学报 2001; 790-792.

33. 李明. 大剂量质子泵抑制剂和 H_2-受体拮抗剂治疗上消化道出血 86 例. 临床医学 2011; 56-57.

34. Brophy GM, Brackbill ML, Bidwell KL, Brophy DF. Prospective, randomized comparison of lansoprazole suspension, and intermittent intravenous famotidine on gastric pH and acid production in critically ill neurosurgical patients. Neurocrit Care 2010; 13: 176-181.

35. Kim YJ, Cheon JH, Lee SK et al. Rebamipide may be comparable to H2 receptor antagonist in healing iatrogenic gastric ulcers created by endoscopic mucosal resection: a prospective randomized pilot study. J Korean Med Sci 2010; 25: 583-588.

36. Furuta T, Shirai N, Sugimoto M et al. Effect of concomitant dosing of famotidine with lansoprazole on gastric acid secretion in relation to CYP2C19 genotype status. Aliment Pharmacol Ther 2005; 22: 67-74.

37. Arbel Y, Birati EY, Finkelstein A et al. Platelet inhibitory effect of clopidogrel in patients treated with omeprazole, pantoprazole, and famotidine: a prospective, randomized, crossover study. Clin Cardiol 2013; 36: 342-346.

38. Nagata Y, Inomata J, Kinoshita M et al. Impact of proton pump inhibitors or famotidine on the antiplatelet actions during dual-antiplatelet therapy in Japanese

patients. Cardiovasc Interv Ther 2013; 28: 22-29.

39. Tunggal P, Ng FH, Lam KF et al. Effect of esomeprazole versus famotidine on platelet inhibition by clopidogrel: a double-blind, randomized trial. Am Heart J 2011; 162: 870-874.

40. 李阳, 王芝荣. 法莫替丁与雷贝拉唑对冠状动脉内支架术后氯吡格雷抗血小板功能影响的比较. 陕西医学杂志 2013; 1186-1188.

41. Nishino M, Sugimoto M, Kodaira C et al. Preventive effects of lansoprazole and famotidine on gastric mucosal injury induced by low-dose aspirin in Helicobacter pylori-negative healthy volunteers. J Clin Pharmacol 2011; 51: 1079-1086.

42. Tolbert K, Bissett S, King A et al. Efficacy of oral famotidine and 2 omeprazole formulations for the control of intragastric pH in dogs. J Vet Intern Med 2011; 25: 47-54.

43. Takahashi Y, Amano Y, Yuki T et al. Influence of acid suppressants on gastric emptying: cross-over analysis in healthy volunteers. J Gastroenterol Hepatol 2006; 21: 1664-1668.

44. Bersenas AM, Mathews KA, Allen DG, Conlon PD. Effects of ranitidine, famotidine, pantoprazole, and omeprazole on intragastric pH in dogs. Am J Vet Res 2005; 66: 425-431.

45. Parkman HP, Urbain JL, Knight LC et al. Effect of gastric acid suppressants on human gastric motility. Gut 1998; 42: 243-250.

46. Iida H, Inamori M, Akimoto K et al. Early effects of intravenous administrations of lansoprazole and famotidine on intragastric pH. Hepatogastroenterology 2009; 56:

551-554.

47. Sakaguchi M, Ashida K, Umegaki E et al. Suppressive action of lansoprazole on gastric acidity and its clinical effect in patients with gastric ulcers: comparison with famotidine. J Clin Gastroenterol 1995; 20: S27-31.
