# Supplementary material for: PPI versus Histamine H2 Receptor Antagonists for Prevention of Upper Gastrointestinal Injury Associated with Low-Dose Aspirin: Systematic Review and Meta-analysis
Source: PLoS One. 2015 Jul 6;10(7):e0131558. doi: 10.1371/journal.pone.0131558 (PMC4493004; doi:10.1371/journal.pone.0131558)
Supplement: S2 File — (PDF) [file pone.0131558.s002.pdf]

Preferred Reporting Items for Systematic Reviews and Meta-Analyses: The PRISMA Statement

David Moher[1,2]*, Alessandro Liberati[3,4], Jennifer Tetzlaff[1], Douglas G. Altman[5], The PRISMA Group¶

1 Ottawa Methods Centre, Ottawa Hospital Research Institute, Ottawa, Ontario, Canada, **2** Department of Epidemiology and Community Medicine, Faculty of Medicine, University of Ottawa, Ottawa, Ontario, Canada, **3** Università di Modena e Reggio Emilia, Modena, Italy, **4** Centro Cochrane Italiano, Istituto Ricerche Farmacologiche Mario Negri, Milan, Italy, **5** Centre for Statistics in Medicine, University of Oxford, Oxford, United Kingdom

Introduction

Systematic reviews and meta-analyses have become increasingly important in health care. Clinicians read them to keep up to date with their field [1,2], and they are often used as a starting point for developing clinical practice guidelines. Granting agencies may require a systematic review to ensure there is justification for further research [3], and some health care journals are moving in this direction [4]. As with all research, the value of a systematic review depends on what was done, what was found, and the clarity of reporting. As with other publications, the reporting quality of systematic reviews varies, limiting readers' ability to assess the strengths and weaknesses of those reviews.

Several early studies evaluated the quality of review reports. In 1987, Mulrow examined 50 review articles published in four leading medical journals in 1985 and 1986 and found that none met all eight explicit scientific criteria, such as a quality assessment of included studies [5]. In 1987, Sacks and colleagues [6] evaluated the adequacy of reporting of 83 meta-analyses on 23 characteristics in six domains. Reporting was generally poor; between one and 14 characteristics were adequately reported (mean = 7.7; standard deviation = 2.7). A 1996 update of this study found little improvement [7].

In 1996, to address the suboptimal reporting of meta-analyses, an international group developed a guidance called the QUOROM Statement (*QU*ality *O*f *R*eporting *O*f *M*eta-analyses), which focused on the reporting of meta-analyses of randomized controlled trials [8]. In this article, we summarize a revision of these guidelines, renamed PRISMA (Preferred Reporting Items for Systematic reviews and Meta-Analyses), which have been updated to address several conceptual and practical advances in the science of systematic reviews (Box 1).

Terminology

The terminology used to describe a systematic review and meta-analysis has evolved over time. One reason for changing the name from QUOROM to PRISMA was the desire to encompass both systematic reviews and meta-analyses. We have adopted the definitions used by the Cochrane Collaboration [9]. A systematic review is a review of a clearly formulated question that uses systematic and explicit methods to identify, select, and critically appraise relevant research, and to collect and analyze data from the studies that are included in the review. Statistical methods (meta-analysis) may or may not be used to analyze and summarize the results of the included studies. Meta-analysis refers to the use of statistical techniques in a systematic review to integrate the results of included studies.

Developing the PRISMA Statement

A three-day meeting was held in Ottawa, Canada, in June 2005 with 29 participants, including review authors, methodologists, clinicians, medical editors, and a consumer. The objective of the Ottawa meeting was to revise and expand the QUOROM checklist and flow diagram, as needed.

The executive committee completed the following tasks, prior to the meeting: a systematic review of studies examining the quality of reporting of systematic reviews, and a comprehensive literature search to identify methodological and other articles that might inform the meeting, especially in relation to modifying checklist items. An international survey of review authors, consumers, and groups commissioning or using systematic reviews and meta-analyses was completed, including the International Network of Agencies for Health Technology Assessment (INAHTA) and the Guidelines International Network (GIN). The survey aimed to ascertain views of QUOROM, including the merits of the existing checklist items. The results of these activities were presented during the meeting and are summarized on the PRISMA Web site (http://www.prisma-statement.org/).

Only items deemed essential were retained or added to the checklist. Some additional items are nevertheless desirable, and review authors should include these, if relevant [10]. For example, it is useful to indicate whether the systematic review is an update [11] of a previous review, and to describe any changes in procedures from those described in the original protocol.

Citation: Moher D, Liberati A, Tetzlaff J, Altman DG, The PRISMA Group (2009) Preferred Reporting Items for Systematic Reviews and Meta-Analyses: The PRISMA Statement. PLoS Med 6(7): e1000097. doi:10.1371/journal.pmed.1000097

Published July 21, 2009

Copyright: © 2009 Moher et al. This is an open-access article distributed under the terms of the Creative Commons Attribution License, which permits unrestricted use, distribution, and reproduction in any medium, provided the original author and source are credited.

Funding: PRISMA was funded by the Canadian Institutes of Health Research; Università di Modena e Reggio Emilia, Italy; Cancer Research UK; Clinical Evidence BMJ Knowledge; the Cochrane Collaboration; and GlaxoSmithKline, Canada. AL is funded, in part, through grants of the Italian Ministry of University (COFIN - PRIN 2002 prot. 2002061749 and COFIN - PRIN 2006 prot. 2006062298). DGA is funded by Cancer Research UK. DM is funded by a University of Ottawa Research Chair. None of the sponsors had any involvement in the planning, execution, or write-up of the PRISMA documents. Additionally, no funder played a role in drafting the manuscript.

Competing Interests: The authors have declared that no competing interests exist.

Abbreviations: PRISMA, Preferred Reporting Items for Systematic reviews and Meta-Analyses; QUOROM, *QU*ality *O*f *R*eporting *O*f *M*eta-analyses.

* E-mail: dmoher@ohri.ca

¶ Membership of the PRISMA Group is provided in the Acknowledgments.

Provenance: Not commissioned; externally peer reviewed. In order to encourage dissemination of the PRISMA Statement, this article is freely accessible on the *PLoS Medicine* Web site (http://medicine.plosjournals.org/) and will be also published in the *Annals of Internal Medicine*, *BMJ*, *Journal of Clinical Epidemiology*, and *Open Medicine*. The authors jointly hold the copyright of this article. For details on further use, see the PRISMA Web site (http://www.prisma-statement.org/).

Box 1: Conceptual Issues in the Evolution from QUOROM to PRISMA

Completing a Systematic Review Is an Iterative Process The conduct of a systematic review depends heavily on the scope and quality of included studies: thus systematic reviewers may need to modify their original review protocol during its conduct. Any systematic review reporting guideline should recommend that such changes can be reported and explained without suggesting that they are inappropriate. The PRISMA Statement (Items 5, 11, 16, and 23) acknowledges this iterative process. Aside from Cochrane reviews, all of which should have a protocol, only about 10% of systematic reviewers report working from a protocol [22]. Without a protocol that is publicly accessible, it is difficult to judge between appropriate and inappropriate modifications.

Conduct and Reporting Research Are Distinct Concepts This distinction is, however, less straightforward for systematic reviews than for assessments of the reporting of an individual study, because the reporting and conduct of systematic reviews are, by nature, closely intertwined. For example, the failure of a systematic review to report the assessment of the risk of bias in included studies may be seen as a marker of poor conduct, given the importance of this activity in the systematic review process [37].

Study-Level Versus Outcome-Level Assessment of Risk of Bias For studies included in a systematic review, a thorough assessment of the risk of bias requires both a "study-level" assessment (e.g., adequacy of allocation concealment) and, for some features, a newer approach called "outcome-level" assessment. An outcome-level assessment involves evaluating the reliability and validity of the data for each important outcome by determining the methods used to assess them in each individual study [38]. The quality of evidence may differ across outcomes, even within a study, such as between a primary efficacy outcome, which is likely to be very carefully and systematically measured, and the assessment of serious harms [39], which may rely on spontaneous reports by investigators. This information should be reported to allow an explicit assessment of the extent to which an estimate of effect is correct [38].

Importance of Reporting Biases Different types of reporting biases may hamper the conduct and interpretation of systematic reviews. Selective reporting of complete studies (e.g., publication bias) [28] as well as the more recently empirically demonstrated "outcome reporting bias" within individual studies [40,41] should be considered by authors when conducting a systematic review and reporting its results. Though the implications of these biases on the conduct and reporting of systematic reviews themselves are unclear, some previous research has identified that selective outcome reporting may occur also in the context of systematic reviews [42].

Shortly after the meeting a draft of the PRISMA checklist was circulated to the group, including those invited to the meeting but unable to attend. A disposition file was created containing comments and revisions from each respondent, and the checklist was subsequently revised 11 times. The group approved the checklist, flow diagram, and this summary paper.

Although no direct evidence was found to support retaining or adding some items, evidence from other domains was believed to be relevant. For example, Item 5 asks authors to provide registration information about the systematic review, including a registration number, if available. Although systematic review registration is not yet widely available [12,13], the participating journals of the International Committee of Medical Journal Editors (ICMJE) [14] now require all clinical trials to be registered in an effort to increase transparency and accountability [15]. Those aspects are also likely to benefit systematic reviewers, possibly reducing the risk of an excessive number of reviews addressing the same question [16,17] and providing greater transparency when updating systematic reviews.

The PRISMA Statement

The PRISMA Statement consists of a 27-item checklist (Table 1; see also Text S1 for a downloadable Word template for researchers to re-use) and a four-phase flow diagram (Figure 1; see also Figure S1 for a downloadable Word template for researchers to re-use). The aim of the PRISMA Statement is to help authors improve the reporting of systematic reviews and meta-analyses. We have focused on randomized trials, but PRISMA can also be used as a basis for reporting systematic reviews of other types of research, particularly evaluations of interventions. PRISMA may also be useful for critical appraisal of published systematic reviews. However, the PRISMA checklist is not a quality assessment instrument to gauge the quality of a systematic review.

From QUOROM to PRISMA

The new PRISMA checklist differs in several respects from the QUOROM checklist, and the substantive specific changes are highlighted in Table 2. Generally, the PRISMA checklist "decouples" several items present in the QUOROM checklist and, where applicable, several checklist items are linked to improve consistency across the systematic review report.

The flow diagram has also been modified. Before including studies and providing reasons for excluding others, the review team must first search the literature. This search results in records. Once these records have been screened and eligibility criteria applied, a smaller number of articles will remain. The number of included articles might be smaller (or larger) than the number of studies, because articles may report on multiple studies and results from a particular study may be published in several articles. To capture this information, the PRISMA flow diagram now requests information on these phases of the review process.

Endorsement

The PRISMA Statement should replace the QUOROM Statement for those journals that have endorsed QUOROM. We hope that other journals will support PRISMA; they can do so by registering on the PRISMA Web site. To underscore to authors, and others, the importance of transparent reporting of systematic reviews, we encourage supporting journals to reference the PRISMA Statement and include the PRISMA Web address in their Instructions to Authors. We also invite editorial organizations to consider endorsing PRISMA and encourage authors to adhere to its principles.

The PRISMA Explanation and Elaboration Paper

In addition to the PRISMA Statement, a supporting Explanation and Elaboration document has been produced [18] following the style used for other reporting guidelines [19–21]. The process

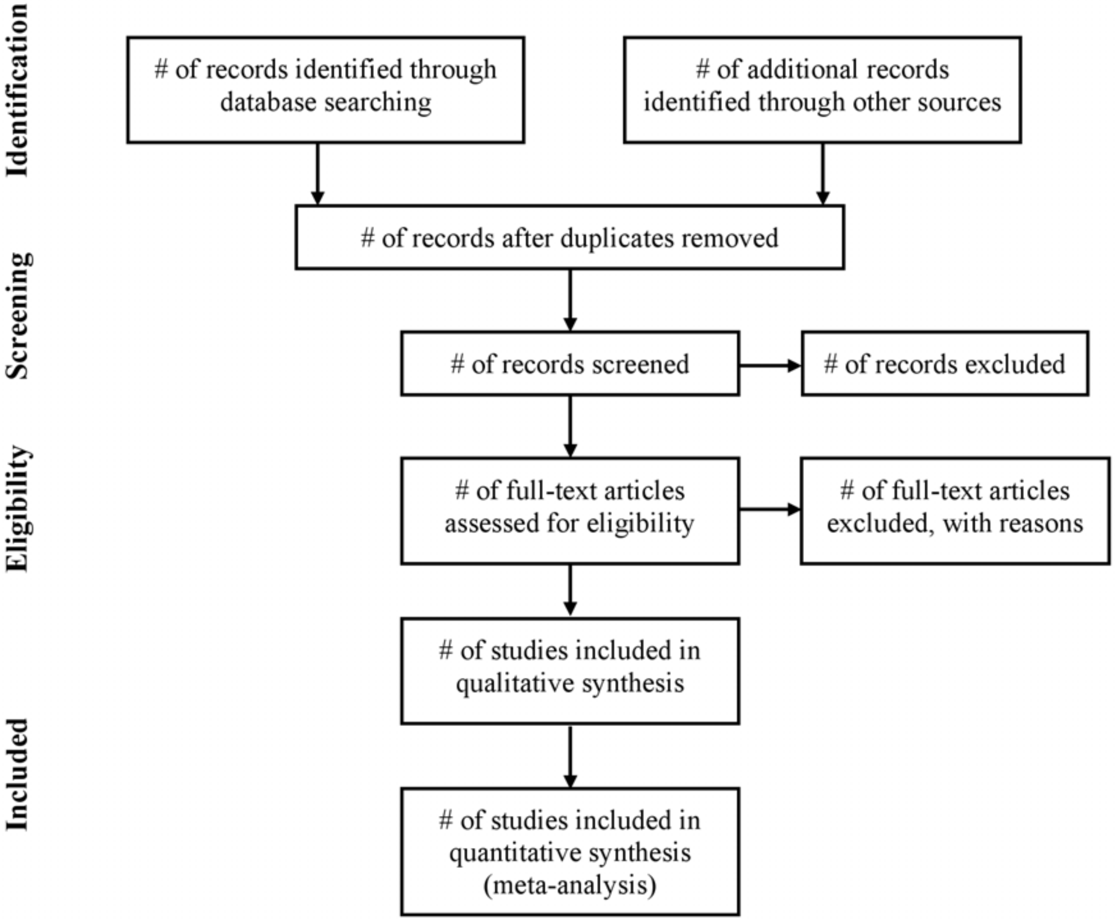

Figure 1. Flow of information through the different phases of a systematic review.
doi:10.1371/journal.pmed.1000097.g001

of completing this document included developing a large database of exemplars to highlight how best to report each checklist item, and identifying a comprehensive evidence base to support the inclusion of each checklist item. The Explanation and Elaboration document was completed after several face to face meetings and numerous iterations among several meeting participants, after which it was shared with the whole group for additional revisions and final approval. Finally, the group formed a dissemination subcommittee to help disseminate and implement PRISMA.

Discussion

The quality of reporting of systematic reviews is still not optimal [22–27]. In a recent review of 300 systematic reviews, few authors reported assessing possible publication bias [22], even though there is overwhelming evidence both for its existence [28] and its impact on the results of systematic reviews [29]. Even when the possibility of publication bias is assessed, there is no guarantee that systematic reviewers have assessed or interpreted it appropriately [30]. Although the absence of reporting such an assessment does not necessarily indicate that it was not done, reporting an assessment of possible publication bias is likely to be a marker of the thoroughness of the conduct of the systematic review.

Several approaches have been developed to conduct systematic reviews on a broader array of questions. For example, systematic reviews are now conducted to investigate cost-effectiveness [31], diagnostic [32] or prognostic questions [33], genetic associations [34], and policy making [35]. The general concepts and topics covered by PRISMA are all relevant to any systematic review, not just those whose objective is to summarize the benefits and harms of a health care intervention. However, some modifications of the checklist items or flow diagram will be necessary in particular circumstances. For example, assessing the risk of bias is a key concept, but the items used to assess this in a diagnostic review are likely to focus on issues such as the spectrum of patients and the verification of disease status, which differ from reviews of interventions. The flow diagram will also need adjustments when reporting individual patient data meta-analysis [36].

We have developed an explanatory document [18] to increase the usefulness of PRISMA. For each checklist item, this document contains an example of good reporting, a rationale for its inclusion, and supporting evidence, including references, whenever possible. We believe this document will also serve as a useful resource for those teaching systematic review methodology. We encourage journals to include reference to the explanatory document in their Instructions to Authors.

Like any evidence-based endeavor, PRISMA is a living document. To this end we invite readers to comment on the revised version, particularly the new checklist and flow diagram, through the PRISMA Web site. We will use such information to inform PRISMA's continued development.

Table 1. Checklist of items to include when reporting a systematic review or meta-analysis.

Section/Topic	#	Checklist Item	Reported on Page #
**TITLE**			
Title	1	Identify the report as a systematic review, meta-analysis, or both.	
**ABSTRACT**			
Structured summary	2	Provide a structured summary including, as applicable: background; objectives; data sources; study eligibility criteria, participants, and interventions; study appraisal and synthesis methods; results; limitations; conclusions and implications of key findings; systematic review registration number.	
**INTRODUCTION**			
Rationale	3	Describe the rationale for the review in the context of what is already known.	
Objectives	4	Provide an explicit statement of questions being addressed with reference to participants, interventions, comparisons, outcomes, and study design (PICOS).	
**METHODS**			
Protocol and registration	5	Indicate if a review protocol exists, if and where it can be accessed (e.g., Web address), and, if available, provide registration information including registration number.	
Eligibility criteria	6	Specify study characteristics (e.g., PICOS, length of follow-up) and report characteristics (e.g., years considered, language, publication status) used as criteria for eligibility, giving rationale.	
Information sources	7	Describe all information sources (e.g., databases with dates of coverage, contact with study authors to identify additional studies) in the search and date last searched.	
Search	8	Present full electronic search strategy for at least one database, including any limits used, such that it could be repeated.	
Study selection	9	State the process for selecting studies (i.e., screening, eligibility, included in systematic review, and, if applicable, included in the meta-analysis).	
Data collection process	10	Describe method of data extraction from reports (e.g., piloted forms, independently, in duplicate) and any processes for obtaining and confirming data from investigators.	
Data items	11	List and define all variables for which data were sought (e.g., PICOS, funding sources) and any assumptions and simplifications made.	
Risk of bias in individual studies	12	Describe methods used for assessing risk of bias of individual studies (including specification of whether this was done at the study or outcome level), and how this information is to be used in any data synthesis.	
Summary measures	13	State the principal summary measures (e.g., risk ratio, difference in means).	
Synthesis of results	14	Describe the methods of handling data and combining results of studies, if done, including measures of consistency (e.g., I^2) for each meta-analysis.	
Risk of bias across studies	15	Specify any assessment of risk of bias that may affect the cumulative evidence (e.g., publication bias, selective reporting within studies).	
Additional analyses	16	Describe methods of additional analyses (e.g., sensitivity or subgroup analyses, meta-regression), if done, indicating which were pre-specified.	
**RESULTS**			
Study selection	17	Give numbers of studies screened, assessed for eligibility, and included in the review, with reasons for exclusions at each stage, ideally with a flow diagram.	
Study characteristics	18	For each study, present characteristics for which data were extracted (e.g., study size, PICOS, follow-up period) and provide the citations.	
Risk of bias within studies	19	Present data on risk of bias of each study and, if available, any outcome-level assessment (see Item 12).	
Results of individual studies	20	For all outcomes considered (benefits or harms), present, for each study: (a) simple summary data for each intervention group and (b) effect estimates and confidence intervals, ideally with a forest plot.	
Synthesis of results	21	Present results of each meta-analysis done, including confidence intervals and measures of consistency.	
Risk of bias across studies	22	Present results of any assessment of risk of bias across studies (see Item 15).	
Additional analysis	23	Give results of additional analyses, if done (e.g., sensitivity or subgroup analyses, meta-regression [see Item 16]).	
**DISCUSSION**			
Summary of evidence	24	Summarize the main findings including the strength of evidence for each main outcome; consider their relevance to key groups (e.g., health care providers, users, and policy makers).	
Limitations	25	Discuss limitations at study and outcome level (e.g., risk of bias), and at review level (e.g., incomplete retrieval of identified research, reporting bias).	
Conclusions	26	Provide a general interpretation of the results in the context of other evidence, and implications for future research.	
**FUNDING**			
Funding	27	Describe sources of funding for the systematic review and other support (e.g., supply of data); role of funders for the systematic review.	

doi:10.1371/journal.pmed.1000097.t001

Table 2. Substantive specific changes between the QUOROM checklist and the PRISMA checklist (a tick indicates the presence of the topic in QUOROM or PRISMA).

Section/Topic	Item	QUOROM	PRISMA	Comment
Abstract		√	√	QUOROM and PRISMA ask authors to report an abstract. However, PRISMA is not specific about format.
Introduction	Objective		√	This new item (4) addresses the explicit question the review addresses using the PICO reporting system (which describes the participants, interventions, comparisons, and outcome(s) of the systematic review), together with the specification of the type of study design (PICOS); the item is linked to Items 6, 11, and 18 of the checklist.
Methods	Protocol		√	This new item (5) asks authors to report whether the review has a protocol and if so how it can be accessed.
Methods	Search	√	√	Although reporting the search is present in both QUOROM and PRISMA checklists, PRISMA asks authors to provide a full description of at least one electronic search strategy (Item 8). Without such information it is impossible to repeat the authors' search.
Methods	Assessment of risk of bias in included studies	√	√	Renamed from "quality assessment" in QUOROM. This item (12) is linked with reporting this information in the results (Item 19). The new concept of "outcome-level" assessment has been introduced.
Methods	Assessment of risk of bias across studies		√	This new item (15) asks authors to describe any assessments of risk of bias in the review, such as selective reporting within the included studies. This item is linked with reporting this information in the results (Item 22).
Discussion		√	√	Although both QUOROM and PRISMA checklists address the discussion section, PRISMA devotes three items (24–26) to the discussion. In PRISMA the main types of limitations are explicitly stated and their discussion required.
Funding			√	This new item (27) asks authors to provide information on any sources of funding for the systematic review.

doi:10.1371/journal.pmed.1000097.t002

Supporting Information

Figure S1 Flow of information through the different phases of a systematic review (downloadable template document for researchers to re-use).
Found at: doi:10.1371/journal.pmed.1000097.s001 (0.08 MB DOC)

Text S1 Checklist of items to include when reporting a systematic review or meta-analysis (downloadable template document for researchers to re-use).
Found at: doi:10.1371/journal.pmed.1000097.s002 (0.04 MB DOC)

Acknowledgments

The following people contributed to the PRISMA Statement: Doug Altman, DSc, Centre for Statistics in Medicine (Oxford, UK); Gerd Antes, PhD, University Hospital Freiburg (Freiburg, Germany); David Atkins, MD, MPH, Health Services Research and Development Service, Veterans Health Administration (Washington, D. C., US); Virginia Barbour, MRCP, DPhil, *PLoS Medicine* (Cambridge, UK); Nick Barrowman, PhD, Children's Hospital of Eastern Ontario (Ottawa, Canada); Jesse A. Berlin, ScD, Johnson & Johnson Pharmaceutical Research and Development (Titusville, New Jersey, US); Jocalyn Clark, PhD, *PLoS Medicine* (at the time of writing, *BMJ*, London, UK); Mike Clarke, PhD, UK Cochrane Centre (Oxford, UK) and School of Nursing and Midwifery, Trinity College (Dublin, Ireland); Deborah Cook, MD, Departments of Medicine, Clinical Epidemiology and Biostatistics, McMaster University (Hamilton, Canada); Roberto D'Amico, PhD, Università di Modena e Reggio Emilia (Modena, Italy) and Centro Cochrane Italiano, Istituto Ricerche Farmacologiche Mario Negri (Milan, Italy); Jonathan J. Deeks, PhD, University of Birmingham (Birmingham, UK); P. J. Devereaux, MD, PhD, Departments of Medicine, Clinical Epidemiology and Biostatistics, McMaster University (Hamilton, Canada); Kay Dickersin, PhD, Johns Hopkins Bloomberg School of Public Health (Baltimore, Maryland, US); Matthias Egger, MD, Department of Social and Preventive Medicine, University of Bern (Bern, Switzerland); Edzard Ernst, MD, PhD, FRCP, FRCP(Edin), Peninsula Medical School (Exeter, UK); Peter C. Gøtzsche, MD, MSc, The Nordic Cochrane Centre (Copenhagen, Denmark); Jeremy Grimshaw, MBChB, PhD, FRCFP, Ottawa Hospital Research Institute (Ottawa, Canada); Gordon Guyatt, MD, Departments of Medicine, Clinical Epidemiology and Biostatistics, McMaster University (Hamilton, Canada); Julian Higgins, PhD, MRC Biostatistics Unit (Cambridge, UK); John P. A. Ioannidis, MD, University of Ioannina Campus (Ioannina, Greece); Jos Kleijnen, MD, PhD, Kleijnen Systematic Reviews Ltd (York, UK) and School for Public Health and Primary Care (CAPHRI), University of Maastricht (Maastricht, Netherlands); Tom Lang, MA, Tom Lang Communications and Training (Davis, California, US); Alessandro Liberati, MD, Università di Modena e Reggio Emilia (Modena, Italy) and Centro Cochrane Italiano, Istituto Ricerche Farmacologiche Mario Negri (Milan, Italy); Nicola Magrini, MD, NHS Centre for the Evaluation of the Effectiveness of Health Care – CeVEAS (Modena, Italy); David McNamee, PhD, *The Lancet* (London, UK); Lorenzo Moja, MD, MSc, Centro Cochrane Italiano, Istituto Ricerche Farmacologiche Mario Negri (Milan, Italy); David Moher, PhD, Ottawa Methods Centre, Ottawa Hospital Research Institute (Ottawa, Canada); Cynthia Mulrow, MD, MSc, *Annals of Internal Medicine* (Philadelphia, Pennsylvania, US); Maryann Napoli, Center for Medical Consumers (New York, New York, US); Andy Oxman, MD, Norwegian Health Services Research Centre (Oslo, Norway); Ba' Pham, MMath, Toronto Health Economics and Technology Assessment Collaborative (Toronto, Canada) (at the time of the first meeting of the group, GlaxoSmithKline Canada, Mississauga, Canada); Drummond Rennie, MD, FRCP, FACP, University of California San Francisco (San Francisco, California, US); Margaret Sampson, MLIS, Children's Hospital of Eastern Ontario (Ottawa, Canada); Kenneth F. Schulz, PhD, MBA, Family Health International (Durham, North Carolina, US); Paul G. Shekelle, MD, PhD, Southern California Evidence Based Practice Center (Santa Monica, California, US); Jennifer Tetzlaff, BSc, Ottawa Methods Centre, Ottawa Hospital Research Institute (Ottawa, Canada); David Tovey, FRCGP, The Cochrane Library, Cochrane Collaboration

(Oxford, UK) (at the time of the first meeting of the group, *BMJ*, London, UK); Peter Tugwell, MD, MSc, FRCPC, Institute of Population Health, University of Ottawa (Ottawa, Canada).

Author Contributions

ICMJE criteria for authorship read and met: DM AL JT DGA. Wrote the first draft of the paper: DM AL DGA. Contributed to the writing of the paper: DM AL JT DGA. Participated in regular conference calls, identified the participants, secured funds, planned the meeting, participated in the meeting, and drafted the manuscript: DM AL DGA. Participated in identifying the evidence base for PRISMA, refining the checklist, and drafting the manuscript: JT. Agree with the recommendations: DM AL JT DGA.

References

1. Oxman AD, Cook DJ, Guyatt GH (1994) Users' guides to the medical literature. VI. How to use an overview. Evidence-Based Medicine Working Group. JAMA 272: 1367–1371.
2. Swingler GH, Volmink J, Ioannidis JP (2003) Number of published systematic reviews and global burden of disease: Database analysis. BMJ 327: 1083–1084.
3. Canadian Institutes of Health Research (2006) Randomized controlled trials registration/application checklist (12/2006). Available: http://www.cihr-irsc.gc.ca/e/documents/rct_reg_e.pdf. Accessed 19 May 2009.
4. Young C, Horton R (2005) Putting clinical trials into context. Lancet 366: 107.
5. Mulrow CD (1987) The medical review article: State of the science. Ann Intern Med 106: 485–488.
6. Sacks HS, Berrier J, Reitman D, Ancona-Berk VA, Chalmers TC (1987) Meta-analysis of randomized controlled trials. New Engl J Med 316: 450–455.
7. Sacks HS, Reitman D, Pagano D, Kupelnick B (1996) Meta-analysis: An update. Mt Sinai J Med 63: 216–224.
8. Moher D, Cook DJ, Eastwood S, Olkin I, Rennie D, et al. (1994) Improving the quality of reporting of meta-analysis of randomized controlled trials: The QUOROM statement. Lancet 354: 1896–1900.
9. Green S, Higgins J, eds (2005) Glossary. Cochrane handbook for systematic reviews of interventions 4.2.5. The Cochrane Collaboration. Available: http://www.cochrane.org/resources/glossary.htm. Accessed 19 May 2009.
10. Strech D, Tilburt J (2008) Value judgments in the analysis and synthesis of evidence. J Clin Epidemiol 61: 521–524.
11. Moher D, Tsertsvadze A (2006) Systematic reviews: When is an update an update? Lancet 367: 881–883.
12. University of York (2009) Centre for Reviews and Dissemination. Available: http://www.york.ac.uk/inst/crd/. Accessed 19 May 2009.
13. The Joanna Briggs Institute (2008) Protocols & work in progress. Available: http://www.joannabriggs.edu.au/pubs/systematic_reviews_prot.php. Accessed 19 May 2009.
14. De Angelis C, Drazan JM, Frizelle FA, Haug C, Hoey J, et al. (2004) Clinical trial registration: A statement from the International Committee of Medical Journal Editors. CMAJ 171: 606–607.
15. Whittington CJ, Kendall T, Fonagy P, Cottrell D, Cotgrove A, et al. (2004) Selective serotonin reuptake inhibitors in childhood depression: Systematic review of published versus unpublished data. Lancet 363: 1341–1345.
16. Bagshaw SM, McAlister FA, Manns BJ, Ghali WA (2006) Acetylcysteine in the prevention of contrast-induced nephropathy: A case study of the pitfalls in the evolution of evidence. Arch Intern Med 166: 161–166.
17. Biondi-Zoccai GG, Lotrionte M, Abbate A, Testa L, Remigi E, et al. (2006) Compliance with QUOROM and quality of reporting of overlapping meta-analyses on the role of acetylcysteine in the prevention of contrast associated nephropathy: Case study. BMJ 332: 202–209.
18. Liberati A, Altman DG, Tetzlaff J, Mulrow C, Gøtzsche P, et al. (2009) The PRISMA statement for reporting systematic reviews and meta-analyses of studies that evaluate health care interventions: Explanation and elaboration. PLoS Med 6: e1000100. doi:10.1371/journal.pmed.1000100.
19. Altman DG, Schulz KR, Moher D, Egger M, Davidoff F, et al. (2001) The revised CONSORT statement for reporting randomized trials: Explanation and elaboration. Ann Intern Med 134: 663–694.
20. Bossuyt PM, Reitsma JB, Bruns DE, Gatsonis CA, Glasziou PP, et al. (2003) Towards complete and accurate reporting of studies of diagnostic accuracy: The STARD explanation and elaboration. Ann Intern Med 138: W1–W12.
21. Vandenbroucke JP, von Elm E, Altman DG, Gøtzsche PC, Mulrow CD, et al. (2007) Strengthening the Reporting of Observational Studies in Epidemiology (STROBE): Explanation and elaboration. Ann Intern Med 147: W163–W194.
22. Moher D, Tetzlaff J, Tricco AC, Sampson M, Altman DG (2007) Epidemiology and reporting characteristics of systematic reviews. PLoS Med 4: e78. doi:10.1371/journal.pmed.0040078.
23. Bhandari M, Morrow F, Kulkarni AV, Tornetta P (2001) Meta-analyses in orthopaedic surgery: A systematic review of their methodologies. J Bone Joint Surg Am 83-A: 15–24.
24. Kelly KD, Travers A, Dorgan M, Slater L, Rowe BH (2001) Evaluating the quality of systematic reviews in the emergency medicine literature. Ann Emerg Med 38: 518–526.
25. Richards D (2004) The quality of systematic reviews in dentistry. Evid Based Dent 5: 17.
26. Choi PT, Halpern SH, Malik N, Jadad AR, Tramer MR, et al. (2001) Examining the evidence in anesthesia literature: A critical appraisal of systematic reviews. Anesth Analg 92: 700–709.
27. Delaney A, Bagshaw SM, Ferland A, Manns B, Laupland KB (2005) A systematic evaluation of the quality of meta-analyses in the critical care literature. Crit Care 9: R575–R582.
28. Dickersin K (2005) Publication bias: Recognizing the problem, understanding its origins and scope, and preventing harm. In: Rothstein HR, Sutton AJ, Borenstein M, eds. Publication bias in meta-analysis-Prevention, assessment and adjustments. Chichester (UK): John Wiley & Sons. pp 11–33.
29. Sutton AJ (2005) Evidence concerning the consequences of publication and related biases. In: Rothstein HR, Sutton AJ, Borenstein M, eds. Publication bias in meta-analysis-Prevention, assessment and adjustments. Chichester (UK): John Wiley & Sons. pp 175–192.
30. Lau J, Ioannidis JP, Terrin N, Schmid CH, Olkin I (2006) The case of the misleading funnel plot. BMJ 333: 597–600.
31. Ladabaum U, Chopra CL, Huang G, Scheiman JM, Chernew ME, et al. (2001) Aspirin as an adjunct to screening for prevention of sporadic colorectal cancer: A cost-effectiveness analysis. Ann Intern Med 135: 769–781.
32. Deeks JJ (2001) Systematic reviews in health care: Systematic reviews of evaluations of diagnostic and screening tests. BMJ 323: 157–162.
33. Altman DG (2001) Systematic reviews of evaluations of prognostic variables. BMJ 323: 224–228.
34. Ioannidis JP, Ntzani EE, Trikalinos TA, Contopoulos-Ioannidis DG (2001) Replication validity of genetic association studies. Nat Genet 29: 306–309.
35. Lavis J, Davies H, Oxman A, Denis J, Golden-Biddle K, et al. (2005) Towards systematic reviews that inform health care management and policy-making. J Health Serv Res Policy 10: 35–48.
36. Stewart LA, Clarke MJ (1995) Practical methodology of meta-analyses (overviews) using updated individual patient data. Cochrane Working Group. Stat Med 14: 2057–2079.
37. Moja LP, Telaro E, D'Amico R, Moschetti I, Coe L, et al. (2005) Assessment of methodological quality of primary studies by systematic reviews: Results of the metaquality cross sectional study. BMJ 330: 1053–1055.
38. Guyatt GH, Oxman AD, Vist GE, Kunz R, Falck-Ytter Y, et al. (2008) GRADE: An emerging consensus on rating quality of evidence and strength of recommendations. BMJ 336: 924–926.
39. Schunemann HJ, Jaeschke R, Cook DJ, Bria WF, El-Solh AA, et al. (2006) An official ATS statement: Grading the quality of evidence and strength of recommendations in ATS guidelines and recommendations. Am J Respir Crit Care Med 174: 605–614.
40. Chan AW, Hrobjartsson A, Haahr MT, Gøtzsche PC, Altman DG (2004) Empirical evidence for selective reporting of outcomes in randomized trials: Comparison of protocols to published articles. JAMA 291: 2457–2465.
41. Chan AW, Krleza-Jeric K, Schmid I, Altman DG (2004) Outcome reporting bias in randomized trials funded by the Canadian Institutes of Health Research. CMAJ 171: 735–740.
42. Silagy CA, Middleton P, Hopewell S (2002) Publishing protocols of systematic reviews: Comparing what was done to what was planned. JAMA 287: 2831–2834.
